# Some Clinical Experience

**Published:** 1892-04

**Authors:** J. H. Jackson


### SOME CLINICAL EXPERIENCE.

J. H. Jackson, M. D.

Dysmenorrhœa, violent cramping pains in uterine region and deadly sickness at stomach, increasing more and more till vomiting water, mucus, and food would give entire relief—this was the history of all previous attacks. The last one was greatly intensified; the face was deadly pale; *the upper lip was puckered together and drawn upwards, exposing the upper teeth.* "*It feels as though my lip would draw up into my nose*" was the way the patient expressed the symptom.

Reading over Gentry's *Concordance Repertory*, I had come across the symptom on page 62, Vol. II: "Upper lip drawn up, exposing teeth," etc. Camphor.

The remedy had not fixed itself in my memory, but the symptom had; and on looking for the word "drawn" I readily found the above peculiar, hence guiding and characteristic symptom. Accordingly administered Camphor$^{cm}$ (Swan), with the satisfaction of giving immediate relief to a *very* sick patient.

On several previous occasions I had prescribed in vain various remedies: the case growing worse and worse till complete relief would follow from vomiting; the patient always being able to eat a hearty meal within twenty minutes after vomiting.

The attack so readily cured by Camphor was an alarming one. The patient looked like one sinking down to death; the fingers were shriveled, icy cold, icy-cold feet, and cold sweat on face and body.

A dozen or more times at the menstrual period this lady has had some pain, accompanied by the symptom, "My upper lip *feels* as though it would draw up." If Camphor$^{cm}$ was taken at once this sensation would pass away in a few minutes, and then menses would be painless.

I advise every homœopathician to procure at once Gentry's *Concordance Repertory*. It is $12 well expended.

Mr. S. for twenty years has not been able to lean back against a chair, the spine being so sensitive he could not bear any pressure upon it.

Several remedies were prescribed without effecting any improvement.

I had been unable to find the exact symptoms anywhere; but on looking in Gentry, under Generalities and Key-Notes, I found under Pressure the symptom: "Cannot bear pressure on any part; *could not rest back against chair;* obliged to change position often; feet painful from resting on floor." Bapt.

Baptisia$^{50m}$ (Swan) cured this sensitiveness, and with it many other symptoms.

Gentry's *Concordance Repertory* is priceless, and much honor is due the author for giving the profession a work of such value.

